# Visual performance and patient preference with bilateral implantation of an extended depth of focus or combined implantation of an extended depth of focus/trifocal intraocular lens

**DOI:** 10.1007/s10792-024-03030-y

**Published:** 2024-02-14

**Authors:** Jonathan Moore, Jens Østergaard, Florian Kretz

**Affiliations:** 1 Cathedral Eye Clinic, Belfast, Northern Ireland; 2 Hellerup Eye Clinic, Copenhagen, Denmark; 3 Augentagesklinik Rheine, Osnabrücker Straße 233 -235, 48429 Rheine, Germany

**Keywords:** Extended depth of focus, Trifocal IOL, Cataract, Refractive lens exchange

## Abstract

**Purpose:**

Evaluate postoperative visual performance in patients with bilaterally implanted AT LARA or AT LARA/AT LISA tri (Carl Zeiss AG, Jena, Germany) intraocular lenses.

**Methods:**

Multicentered, comparative, open-label, retrospective/prospective study. Post-IOL implantation, patients were prospectively enrolled into this study; preoperative patient data were collected retrospectively. Follow-up was at 2–4 and 5–8 months post-surgery. The primary endpoint was binocular best corrected distance visual acuity (CDVA). The study was retrospectively registered on clinicaltrials.gov (#NCT05462067).

**Results:**

Seventy-one patients (142 eyes) were enrolled; 67 patients (134 eyes) have 5–8 months data. The mean binocular CDVA at 2–4 months was -0.10 ± 0.06 logMAR in the bilateral AT LARA group (“bilateral”) and -0.11 ± 0.09 logMAR in the combined implantation AT LARA/ AT LISA tri group (“combined implantation”); (*P* = 0.4856). At 5–8 months, mean binocular CDVA was -0.13 ± 0.06 logMAR in the bilateral group and -0.11 ± 0.09 in the combined implantation group (*P* = 0.4003). At 5–8 months, more eyes in the bilateral group attained 0.2 logMAR or better binocular uncorrected intermediate VA (UIVA; 67 cm) than those in the combined implantation group (100% vs. 94%, respectively). The bilateral group achieved a mean of 0.24 ± 0.11 logMAR in uncorrected near VA (UCNVA), compared to a mean of 0.16 ± 0.12 logMAR in the combined implantation group at 5–8 months (*P* = 0.0041).

**Conclusions:**

A combined implantation approach (AT LARA in the distance dominant eye/AT LISA tri in the non-dominant eye) produced similar CDVA outcomes but better UCNVA as bilateral implantation with the AT LARA. UIVA was comparable between groups. No new safety concerns were reported.

## Introduction

Multifocal intraocular lenses (MFIOLs) were designed to enhance visual outcomes after cataract surgery by correcting the effects of presbyopia [1–4]. Trifocal IOLs have been shown to provide patients with better vision across all distances, and specifically intermediate vision [1, 2, 5–8], whereas previous research has shown bifocal IOLs are less effective at correcting intermediate vision [1, 2, 4, 6, 9]. In today’s technology-driven world, intermediate vision has become a crucial component of postoperative patient satisfaction. MFIOLs are not without their challenges, however, including glare, halo, loss of contrast sensitivity, and unsatisfactory uncorrected visual acuity [1, 4]. Trifocal lenses can also produce unwanted optical phenomena [1, 4, 8, 9]; this occurs because light energy is split among the rings and directed to different focal points [5, 9].

To mitigate these visual disturbances coupled with the ever-increasing demand for better intermediate vision, extended-depth-of-focus (EDOF) IOLs have been introduced. EDOF lens technology provides continuous range of focus [1, 9–14], leading to fewer unwanted visual disturbances (i.e., photic phenomena, glare, halos) without sacrificing other visual outcomes [10, 11, 15]. Numerous studies to date have shown EDOF IOLs produce similar results in both uncorrected far and intermediate vision as trifocal IOLs [1, 5, 11, 13, 14]. However, trifocal IOLs tend to produce better results at near, with EDOF lenses requiring greater spectacle need [1, 12, 16].

A “combined implantation approach,” where surgeons implant the EDOF IOL in the dominant eye and a trifocal IOL in the nondominant eye, attempts to offer better functional near vision with fewer photic phenomena while continuing to provide excellent intermediate and far results [1, 9].

This study was designed to evaluate the postoperative visual results between bilateral AT LARA 829MP/929 M/MP (Carl Zeiss AG, Jena, Germany), a diffractive EDOF, and a combined implantation approach with the AT LARA and AT LISA tri 839MP/939 M/MP (Carl Zeiss AG), a diffractive trifocal. The primary outcome was postoperative visual performance and patient and surgeon satisfaction in patients implanted with either the non-toric or toric versions of the lenses.

## Methods

This was a multicentered, comparative, open-label, retrospective/prospective noninferiority study; patients were either previously scheduled for cataract surgery or expressed an interest in refractive lens exchange (RLE). The retrospective portion of this study was the real-world clinical assessment of patient eligibility for an EDOF or trifocal lens, as well as the crystalline lens removal and surgical implantation of either the AT LARA or AT LISA tri; both toric and non-toric versions of the lenses were used. Post-IOL implantation, patients became eligible for the prospective component of the study; preoperative patient characteristics and surgical data was collected retrospectively. Key inclusion criteria for the prospective component were: uncomplicated implantation of the study IOLs, no visual acuity (VA)-limiting pathologies, and clear intraocular media. Patients were excluded from the study if any of the following occurred: postoperative visual potential of > 0.2 logMAR in each eye due to ocular pathologies; postoperative corrected distance VA (CDVA) of > 0.2 logMAR; acute or chronic illness (e.g., macular degeneration, cystoid macular edema, proliferative diabetic retinopathy); corneal abnormalities (e.g., forme fruste keratoconus, keratoconus); pupil abnormalities; or use of systemic/ocular medications that could affect vision outcomes.

Planned follow-up was at 2–4 months post-surgery and at 5–8 months post-surgery. The primary endpoint was the comparison of binocular CDVA, measured at both of the follow-up time points, to assess noninferiority between the two approaches.

Secondary outcomes included the postoperative binocular uncorrected distance VA (UDVA); uncorrected intermediate VA (UIVA), measured at 67 cm; uncorrected near VA (UNVA), measured at 40 cm; and sphere, cylinder, and manifest refractive spherical equivalent (MRSE). Defocus curves were obtained binocularly for vergences from + 1.00 to − 4.00 D (in 0.50 D step sizes). VA was determined using suitable VA charts and then converted to logMAR.

Patient-reported parameters were determined via the validated McAlinden questionnaire that addressed spectacle use postoperatively and overall satisfaction. There were additional questions to ascertain surgeon satisfaction rates (these have not been validated; these were added onto the original McAlinden questionnaire). All four centers used the same questionnaire.

Other secondary outcomes included rate and/or percentage of eyes with reported photic phenomena.

This study was carried out in compliance with EN ISO 14155:2012-01 Clinical Investigation of Medical Devices for Human Subjects, German Medical device law (Medical Devices Act—MPG) §23 b, MPSV—Ordinance on the Registration, Evaluation and Prevention of Risks of Medical Devices (Medical Devices Safety Plan Ordinance). The study was conducted in accordance with the ethics principles of the Declaration of Helsinki, and all patients provided informed consent. The study was approved by all local ethics committees and was retrospectively registered on clinicaltrials.gov (#NCT05462067).

### IOLs

Acar et al. [1] have described the technology of the AT LISA tri and the AT LARA in detail. To summarize, both of these IOLs are based on a trifocal optical design, with the AT LARA being aberration-neutral, enabling the spherical aberration of the cornea to elongate each focal point. Further, the AT LISA tri is an aspheric, diffractive lens with + 3.33 D of near add and + 1.66 D of intermediate add at the IOL plane, while the AT LARA is an aspheric, diffractive lens with a depth of focus extension of + 0.95 D and + 1.9 D. The non-toric version of the AT LARA is available from − 10.0 D to + 32.0 D in steps of 0.5 D, whereas the toric version has a spherical equivalent refraction of − 4.0 D to + 32.0 D in steps of 0.5 D, and cylinder of + 1.0 to + 12.0 D in steps of 0.5 D. The AT LISA tri non-toric version is available from 0.0 D to + 32.0 D in steps of 0.5 D, whereas the toric version has a spherical equivalent refraction of − 10.0 to + 32.0 D and cylinder of + 1.0 to + 12.0 D in steps of 0.5 D.

The AT LARA differs from other EDOF lenses by providing a wider range of focus and a patented smooth surface design with shallower angles to minimize light scattering and provide visual comfort at night [1].

### Statistical analysis

Sample size calculation was performed with Sealed Envelope Ltd. 2012.^1^ If there is truly no difference between the standard and experimental treatment, then 138 patients (69 per group) were required to ensure that the lower limit of a two-sided 90% confidence interval will be above the non-inferiority limit of − 0.03. The non-inferiority limit is based on the results of a pilot study in 20 patients. The required sample size was not achieved as designated in the protocol as enrollment was halted early, due both in part to the COVID pandemic and slower-than-anticipated potential eligible patients at each site; a “best case analysis set” was used instead for all statistical analyses.

The remaining data was evaluated using SPSS software for Windows 19.0 (IBM, Armonk, NY, USA). The normality of the data sets is evaluated by the Kolmogorov–Smirnov test. If the data are normally distributed, parametric statistics were used. The Student's t-test for paired samples was used to compare preoperative and postoperative data. The Student's t-test for independent LARA LISA-2018–01—Version 1.0–Jan 02, 2019 samples was used for comparisons between groups. When parametric analysis was not possible, the differences between preoperative and postoperative data were evaluated with the Wilcoxon rank-sum test. The Mann–Whitney *U* test was used for comparisons between groups. For the evaluation of the questionnaire, the Fisher`s exact test was used to evaluate differences in categorical variables between the groups.

For all statistical tests, a *p*-value below 0.05 is considered statistically significant.

## Results

There were a total of 71 patients (142 eyes) enrolled with follow-up data for 2–4 months; 67 patients (134 eyes) were included in the 5–8 month best-case analysis set. A total of four clinical sites were involved in this study; two sites enrolled patients implanted bilaterally with the AT LARA (*n* = 20); 3 sites enrolled patients with the combined implantation AT LARA/AT LISA tri (*n* = 51). One patient underwent cataract surgery in one eye and RLE in the contralateral eye; this subject was excluded from the statistical analysis. One patient was excluded from analysis as the patient had an add-on IOL implanted before the first scheduled follow-up period. Three patients were excluded from the 5–8 month follow-up period as no data were collected, and another patient was excluded from the 5–8 month follow-up due to an adverse event with potentially deleterious effect on vision (retinal detachment). The retinal detachment was determined by the surgeon not to be device-related.

### Patient demographics

The mean age of the bilateral AT LARA patients was 58.6 ± 8.4 years, while the mean age of the combined implantation AT LARA/AT LISA tri patients was 61.8 ± 9.0 years (P = 0.183); the mean age of those undergoing cataract surgery (63.4 ± 9.3 years) and those undergoing RLE (57.3 ± 7.2 years) was statistically significant (P = 0.003; Mann–Whitney *U*-test). More patients were female (*n* = 38); of these female patients, 9 (45%) underwent bilateral AT LARA implantation while 29 (56.9%) underwent combined implantation of the AT LARA/AT LISA tri). A total of 42 patients (60.6%) underwent cataract surgery: 10 patients (52.6%) had bilateral AT LARA implantations, while 32 patients (62.7%) had combined implantation AT LARA/AT LISA tri. Of the 28 patients who underwent RLE, 9 (47.4%) received bilateral AT LARA, and 19 (37.3%) received the combined implantation AT LARA/AT LISA tri. A total of 56 eyes (39.5%) underwent toric lens implantation; 30 eyes in the cataract group and 26 eyes in the RLE group; of these, 14 eyes were implanted with the AT LISA tri toric lens, and 42 eyes were implanted with the AT LARA lens.

One patient in the bilateral AT LARA group was targeted for mini-monovision (with the targeted difference between eyes of 0.25D).

As the prospective part of this study was post-surgical, typical preoperative variables (e.g., IOL power calculations, biometry, lens choice) and surgical technique were at the investigators’ discretion (real-world clinic) and data was collected retrospectively. This study was not designed to evaluate the preoperative parameters, and no between-group comparisons were made.

### Primary outcome: postoperative CDVA

All patients in the combined implantation AT LARA/AT LISA tri group were 0.4 logMAR or better at baseline, compared to 17/18 eyes (94%) of the patients in the bilateral AT LARA group. By the 2–4 month follow-up visit, all patients in the bilateral AT LARA group and 94% of those in the combined implantation AT LARA/AT LISA tri group were at 0.0 logMAR or better. The same percentage of eyes in the two groups (80%) were − 0.1 logMAR. By the 5–8 month follow-up, 96% of those in the combined implantation AT LARA/AT LISA tri group were 0.0 logMAR or better. 95% of those in the bilateral AT LARA group and 81% in the combined implantation AT LARA/AT LISA tri group reached − 0.1 logMAR or better. Table 1 provides the mean and median ranges for binocular CDVA in both groups.

**Table 1:** Pre- and post-operative binocular corrected distance visual acuity

Binocular CDVA [logMAR]				
Timepoints		Preoperative	2–4 M	5–8 M
Bilateral AT LARA	Mean	0.16	− 0.10	− 0.13
	SD	0.16	0.06	0.06
	Median	0.10	− 0.10	− 0.10
	Min	− 0.10	− 0.20	− 0.20
	Max	0.50	0.00	0.00
	Subtotal	**18**	**20**	**19**
Combined implantation AT LARA/ AT LISA tri	Mean	0.06	− 0.11	− 0.11
	SD	0.15	0.09	0.09
	Median	0.10	− 0.10	− 0.10
	Min	− 0.20	− 0.20	− 0.20
	Max	0.40	0.20	0.20
	Subtotal	**45**	**51**	**48**
	*p*–value^a^	0.0492	0.4856	0.4003

Bold highlights the sub-total

*CDVA* corrected distance visual acuity

^a^Mann-Whitney *U*-Test

### Other refractive outcomes

Preoperative and postoperative mean refractive errors are summarized in Table 2. There were no statistically significant differences between the two groups in sphere, cylinder, or MRSE at any time point (Mann–Whitney *U*-Test).

**Table 2:** Mean unilateral refractive errors

	Bilateral AT LARA			AT LARA/AT LISA tri		
	Preop (*n* = 40)	2–4 months (*n* = 40)	5–8 months (*n* = 38)	Preop (*n* = 100)	2–4 months (*n* = 102)	5–8 months (*n* = 96)
Sphere (D) ± SD	− 1.66 ± 3.79	0.15 ± 0.28	0.18 ± 0.22	0.20 ± 3.85	0.24 ± 0.39	0.22 ± 0.36
Cylinder (D) ± SD	− 0.93 ± 0.88	− 0.23 ± 0.28	− 0.27 ± 0.28	− 0/82 ± 0.95	− 0.31 ± 0.3	− 0.28 ± 0.29
Spherical equivalent (D) ± SD	− 2.12 ± 3.9	0.03 ± 0.29	0.04 ± 0.26	− 0.21 ± 3.86	0.08 ± 0.36	0.08 ± 0.31

*Preop* preoperatively, *D* diopter, *SD* standard deviation

### Binocular UDVA

**Fig. 1:**
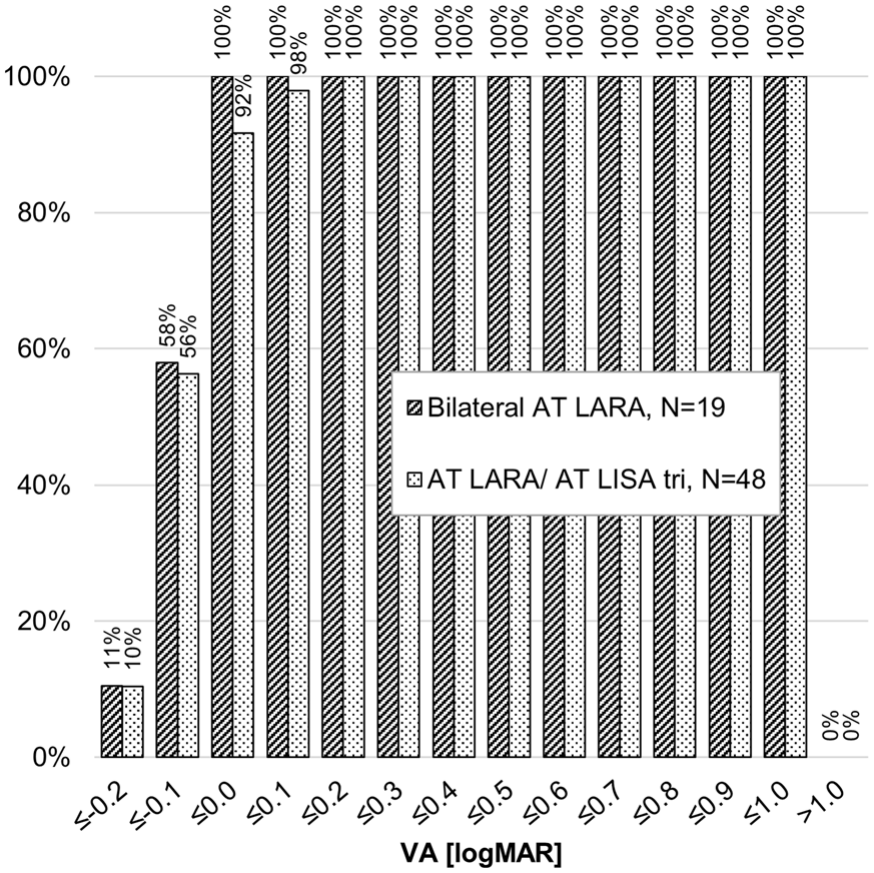
Cumulative binocular uncorrected distance visual acuity at the 5–8 month follow-up. VA, visual acuity; Mann–Whitney U-Test

The postoperative binocular UDVA (logMAR) was similar between groups and not statistically or clinically significant, with the mean in the bilateral AT LARA group of − 0.06 ± 0.07 at the 2–4 month follow-up and − 0.07 ± 0.07 at the 5–8 month follow-up, and the mean in the combined implantation AT LARA/AT LISA tri group of − 0.05 ± 0.09 at the 2–4 month follow-up and − 0.06 ± 0.08 at the 5–8 month follow-up (*P* = 0.8399 at the 2–4 month follow-up and *P* = 0.7181 at the 5–8 month follow-up; Mann–Whitney *U*-Test). At the 5–8 month follow-up, 58% of the LARA/LARA group and 56% of the LARA/LISA group had attained − 0.1 logMAR or better UDVA. See Fig. 1.

### Intermediate visual acuity

**Fig. 2:**
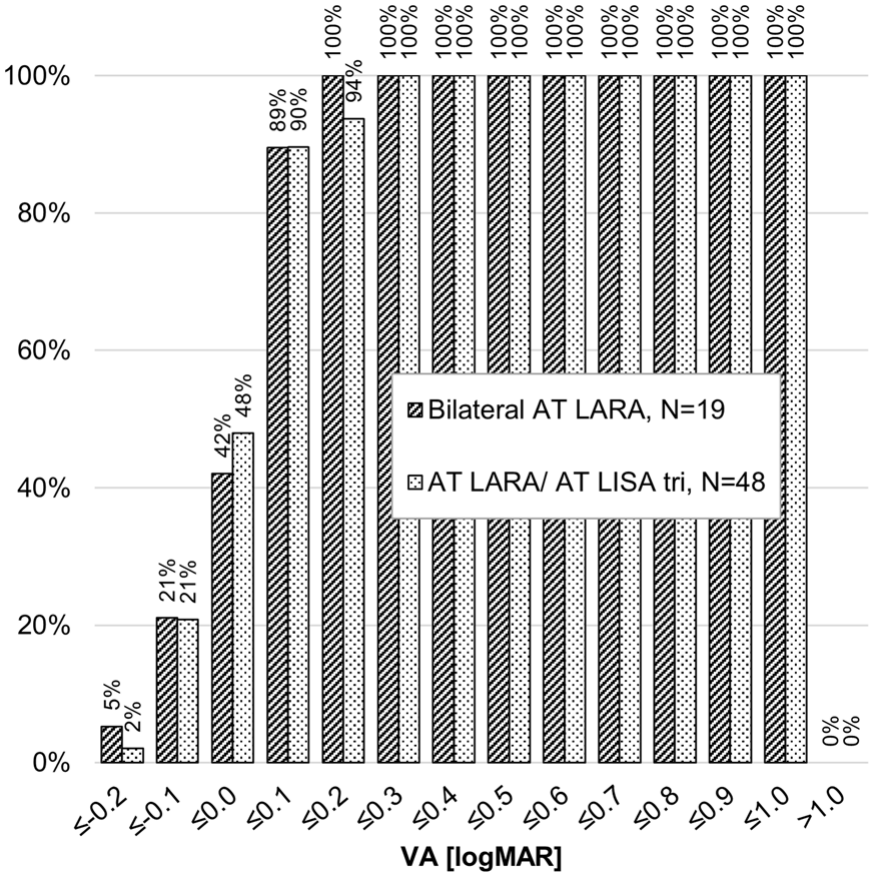
Cumulative binocular uncorrected intermediate visual acuity per study group at the 5–8 month follow-up. VA, visual acuity. Intermediate vision was evaluated at 67 cm

Cumulatively, more eyes in the bilateral AT LARA group attained 0.4 logMAR or better binocular uncorrected intermediate VA (UIVA) than those in the combined implantation AT LARA/AT LISA tri group at 2–4 months (100% vs. 98%, respectively) when evaluated at 67 cm. At 2–4 months, the mean UIVA in the bilateral AT LARA group was − 0.01 ± 0.10 logMAR (min − 0.20, max 0.20) and 0.06 ± 0.12 logMAR (min − 0.20, max 0.47) in the combined implantation AT LARA/ AT LISA tri group; this was statistically significant (*P* = 0.0475; Mann–Whitney *U*-Test). By the 5–8 month follow-up, differences in binocular UIVA were not noticeable until 0.2 logMAR or better (100% vs. 94%, respectively). At 5–8 months, the mean UIVA in the bilateral AT LARA group was 0.02 ± 0.11 logMAR (min − 0.22, max 0.18) and 0.03 ± 0.11 logMAR (min − 0.24, max 0.30) in the combined implantation AT LARA/AT LISA tri group. More eyes in the combined implantation AT LARA/ AT LISA tri group than in the bilateral AT LARA group achieved 0.1–0.0 logMAR (90% vs. 89% and 48% to 42%, respectively). See Fig. 2. There were no differences between the two groups in binocular distance-corrected intermediate VA.

#### Near visual acuity

There was a statistically significant difference between the two groups at both follow-up time points in uncorrected near VA. Table 3 shows both the mean and median binocular UNVA.

**Table 3:** Postoperative binocular uncorrected near visual acuity per study group

Binocular UNVA [logMAR]			
Timepoints		2–4 M	5–8 M
Bilateral AT LARA	Mean	0.26	0.24
	SD	0.15	0.11
	Median	0.20	0.20
	Min	0.00	0.00
	Max	0.70	0.50
	Subtotal	**20**	**19**
Combined implantation AT LARA/AT LISA tri	Mean	0.15	0.16
	SD	0.14	0.12
	Median	0.10	0.10
	Min	0.00	0.00
	Max	0.60	0.60
	Subtotal	**51**	**48**
	*p*–value^a^	0.0049*	0.0041*

Bold highlights the sub-total

*UNVA* uncorrected near visual acuity

^a^Mann-Whitney *U*-Test; *statistically significant (*p* < 0.05)

**Fig. 3:**
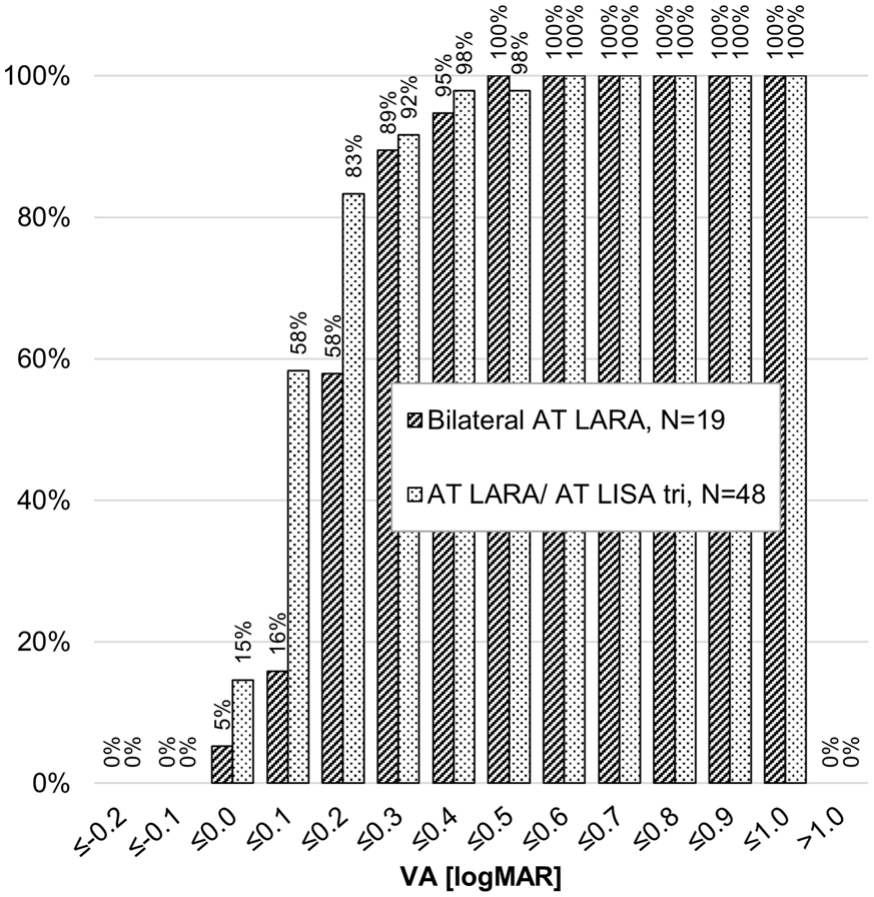
Cumulative binocular uncorrected near visual acuity at 5–8 months. VA, visual acuity

The near vision outcomes heavily favor the combined implantation group at 0.2 logMAR or better, beginning at the 2–4 month follow-up and continuing through the 5–8 month follow-up (see Fig. 3 for the 5–8 month cumulative outcomes).

**Fig. 4:**
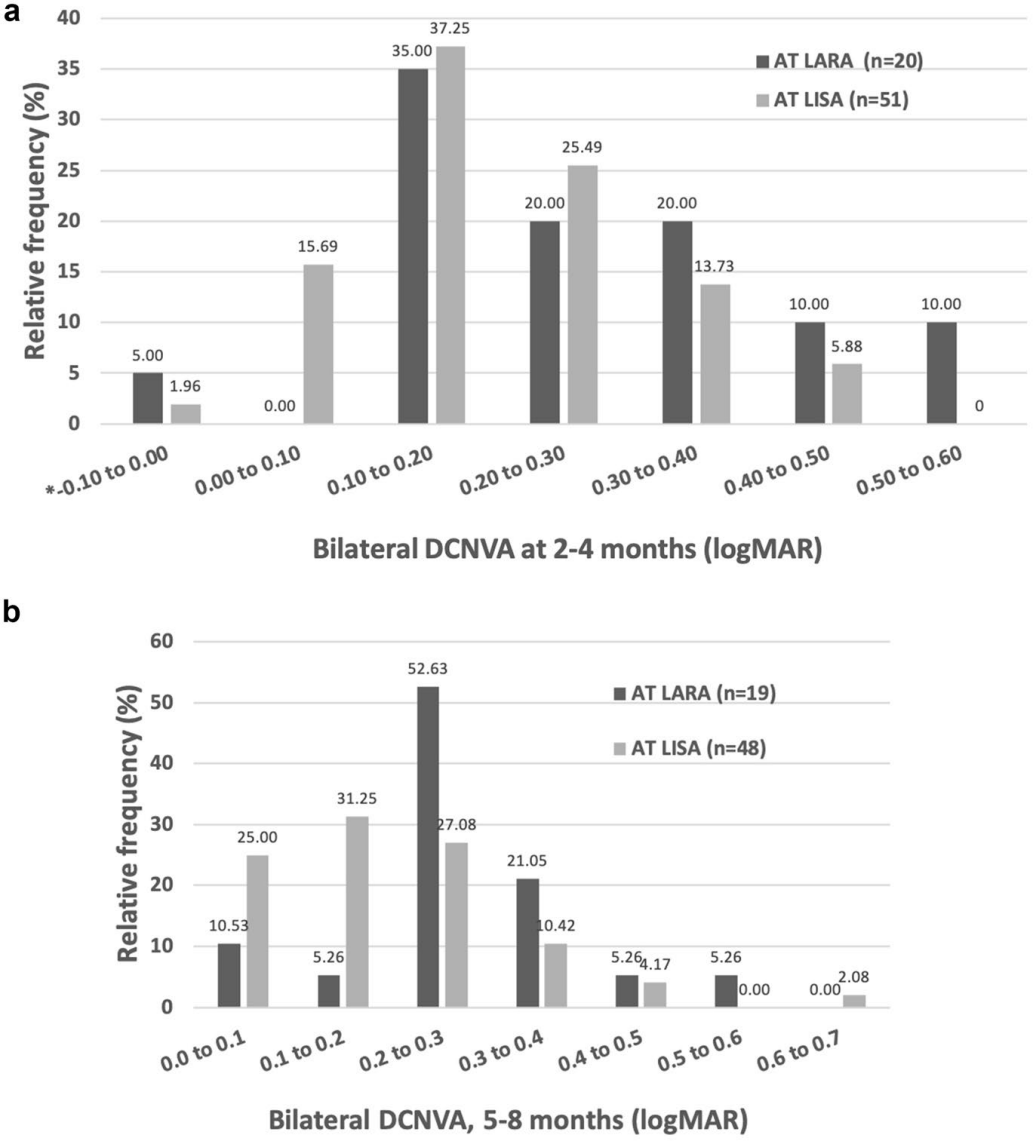
Binocular distance corrected near visual acuity. DCNVA, distance-corrected near visual acuity

There was a statistically significant difference in DCNVA at the 5–8 month follow-up but not at the 2–4 month follow-up. See Fig. 4.

### Spectacle use

Patients were administered a validated questionnaire [17] to assess spectacle use. When asked how often patients needed spectacles for distance vision, 94.4% of the patients in the bilateral AT LARA group and 91.3% in the combined implantation AT LARA/AT LISA tri group answered “none of the time” at the 5–8 month follow-up. One patient in the bilateral AT LARA group (5.3%) and 4 patients in the combined implantation AT LARA/AT LISA tri group (8%) responded “A little of the time” at the 2–4 month follow-up; one of the patients in the bilateral AT LARA group (5.3%) and one in the combined implantation AT LARA/AT LISA tri group (2.2%) needed spectacles “all the time” at the 5–8 month follow-up. Table 4 shows the responses per group at the two follow-up time points. There were no statistically significant differences in spectacle use between the cataract and RLE groups at any distance, regardless of the lens implantation group.

**Table 4:** Postoperative need for glasses in each group at each follow-up time point

How often do you need glasses for … vision?													
		Distance vision				Intermediate vision				Near vision			
Timepoints		2–4 M		5–8 M		2–4 M		5–8 M		2–4 M		5–8 M	
		N	%	N	%	N	%	N	%	N	%	N	%
Bilateral AT LARA	All the time	0	0.0	0	0.0	0	0.0	1	5.6	3	16.7	3	16.7
	Most of the time	0	0.0	0	0.0	1	5.6	1	5.6	6	33.3	4	22.2
	Some of the time	0	0.0	0	0.0	1	5.6	1	5.6	3	16.7	3	16.7
	A little of the time	1	5.6	1	5.6	2	11.1	1	5.6	2	11.1	6	33.3
	None of the time	17	94.4	17	94.4	14	77.8	14	77.8	4	22.2	2	11.1
	Subtotal	18	100	18	100	18	100	18	100	18	100	18	100
*p*-values^a^		0.6547				0.6750				0.6241			
Combined implantation AT LARA/AT LISA tri	All the time	0	0.0	1	2.2	1	2.2	1	2.2	3	6.5	4	8.7
	Most of the time	0	0.0	0	0.0	0	0.0	1	2.2	3	6.5	4	8.7
	Some of the time	0	0.0	1	2.2	3	6.5	2	4.3	6	13.0	6	13.0
	A little of the time	4	8.7	2	4.3	2	4.3	1	2.2	10	21.7	10	21.7
	None of the time	42	91.3	42	91.3	40	87.0	41	89.1	24	52.2	22	47.8
	Subtotal	46	100	46	100	46	100	46	100	46	100	46	100
*p*-values^a^		0.5002				0.7532				0.4513			

^a^Wilcoxon signed-rank test

### Safety

**Fig. 5:**
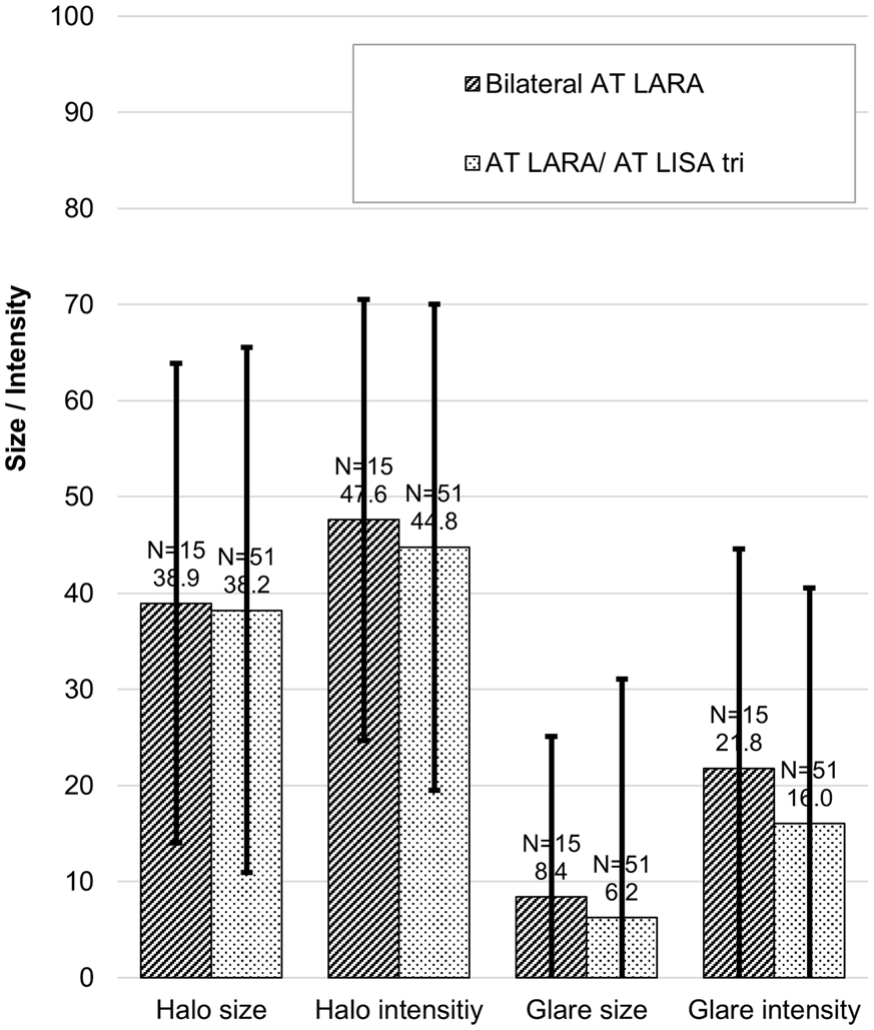
Halo and glare simulator outcomes at the 2–4 month follow-up

**Fig. 6:**
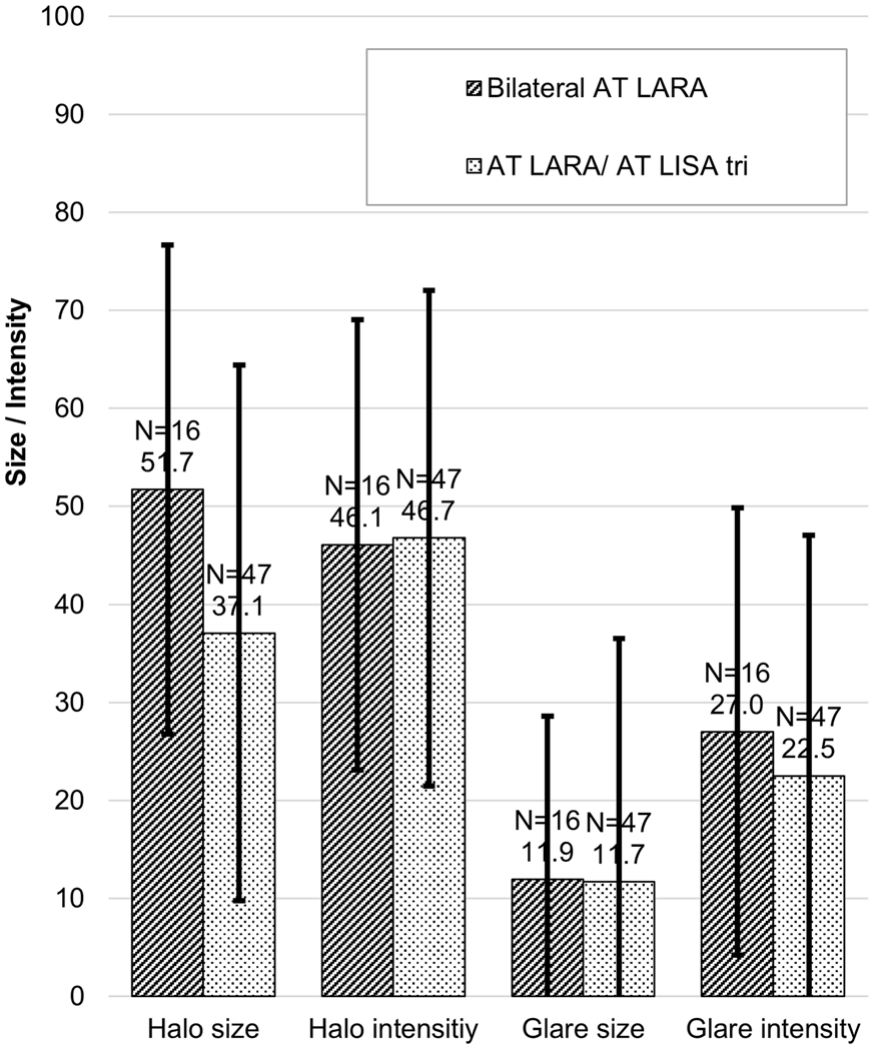
Halo and glare simulator outcomes at the 5–8 month follow-up

Halos were similar between the two groups at 2–4 months but were statistically significantly different in size at the 5–8 month follow-up (51.69 ± 21.82 in the bilateral AT LARA group vs. 37.06 ± 19.02 in the combined implantation AT LARA/ AT LISA tri group; *P* = 0.0125); halo size increased substantially in the bilateral AT LARA group from the 2–4 month time point to the 5–8 month timepoint (38.93 ± 24.8 to 51.69 ± 21.82, respectively), but remained similar in the combined implantation AT LARA/AT LISA tri group (38.24 ± 18.95 to 37.06 ± 19.02, respectively). There were no statistically significant differences between the two groups at any follow-up point for glare. See Figs. 5 and 6 for differences in halo and glare size and intensity at the two follow-up time points.

### Defocus

**Fig. 7:**
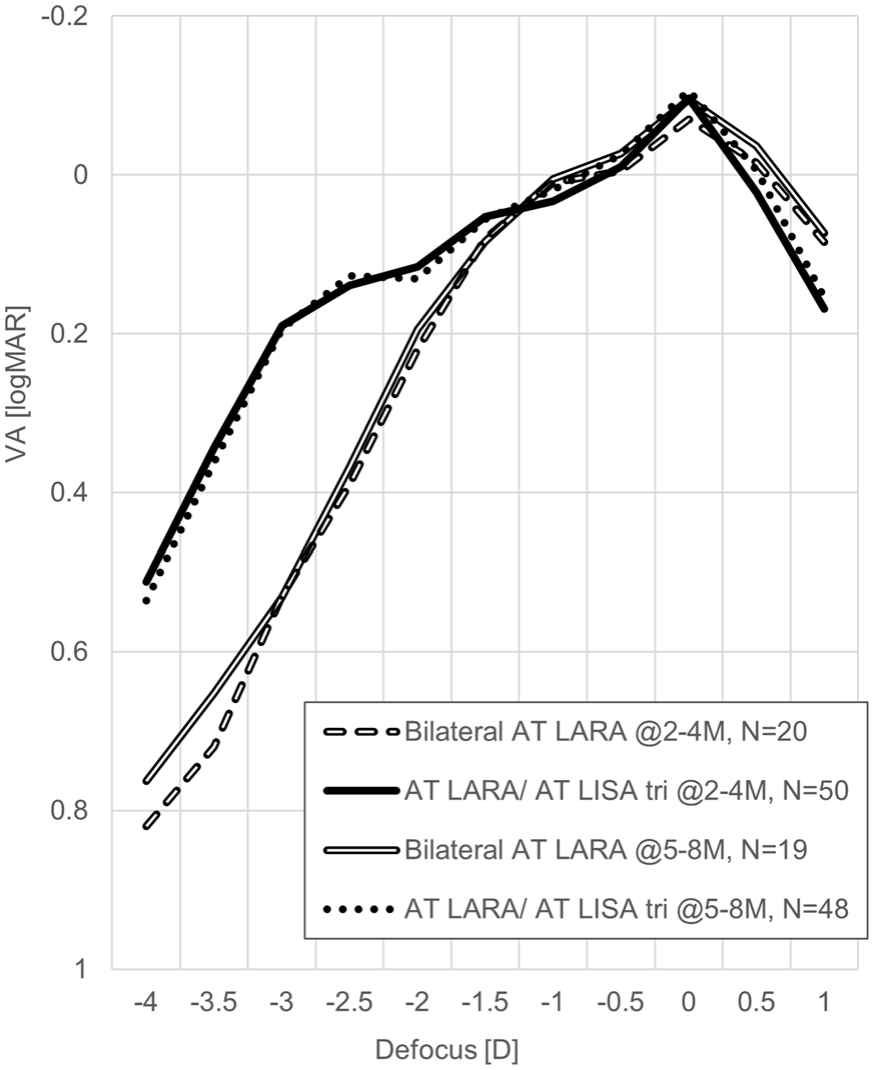
Defocus curves per postoperative visit and per study group

Binocular defocus curves were compared under photopic conditions between groups at both follow-up time points and remained stable throughout the follow-up time points. The combined implantation group had a wider range of focus compared to the bilateral group: A logMAR of 0.2 or better was achieved between − 2.0D and + 1.0D in both groups at all follow-up time points; but the combined implantation group had a logMAR of 0.2 or better from − 3.0 D to + 1.0 D at both follow-up time points. There were statistically significant differences between groups in defocus curves at − 2.5, − 3, − 3.5, − 4, and at 1 at both follow-up time points (*P* < 0.001; Mann–Whitney *U*-Test). Figure 7 shows the comparison between groups in defocus curves at all time points.

### Prediction error

Prediction errors were within acceptable ranges, with the majority of eyes within ± 0.25D at all follow-up time points. At 2–4 months, 57.9% (*n* = 22) of eyes in the bilateral AT LARA group and 52.1% (*n* = 50) of eyes in the combined implantation AT LARA/AT LISA tri group were within 0.25D of target. At 5–8 months, 55% (*n* = 22) of eyes in the bilateral AT LARA group and 52% (*n* = 51) of eyes in the combined implantation AT LARA/AT LISA tri group were within 0.25D of target. All eyes in the bilateral AT LARA group were within 0.75D of target, while all but one eye in the combined implantation AT LARA/ AT LISA tri group were within 1D of target.

### Adverse events

One patient in the combined implantation AT LARA/AT LISA tri group (2.2%) showed bilateral posterior capsule opacification (PCO) requiring treatment in the 2–4 months follow-up visit. A YAG-laser treatment was conducted as secondary intervention. In the 5–8 month follow-up, no PCO was noted. Two eyes in the combined implantation AT LARA/AT LISA tri group (4.3%) developed a retinal detachment; one of those patients developed retinal foramen in the contralateral eye (both at the 2–4 month follow-up); neither of these retinal detachments was device-related. One eye in the bilateral AT LARA group (5.5%) failed to achieve the desired degree of spectacle independence for near before the 2–4 month follow-up and was subsequently implanted with an add-on IOL + 1.25D.

### Patient and surgeon satisfaction

Using the validated McAlinden questionnaire, the majority of patients would recommend a multifocal lens again, with 94.7% in the bilateral AT LARA group (*n* = 18) and 93% in the combined implantation AT LARA/AT LISA tri group (*n* = 40) at the 5–8 month follow-up. By the 5–8 month follow-up, all patients in the bilateral AT LARA group would recommend the same lens to relatives and family, compared to 91.7% (*n* = 44) in the combined implantation AT LARA/AT LISA tri group. These differences were not statistically significant.

**Fig. 8:**
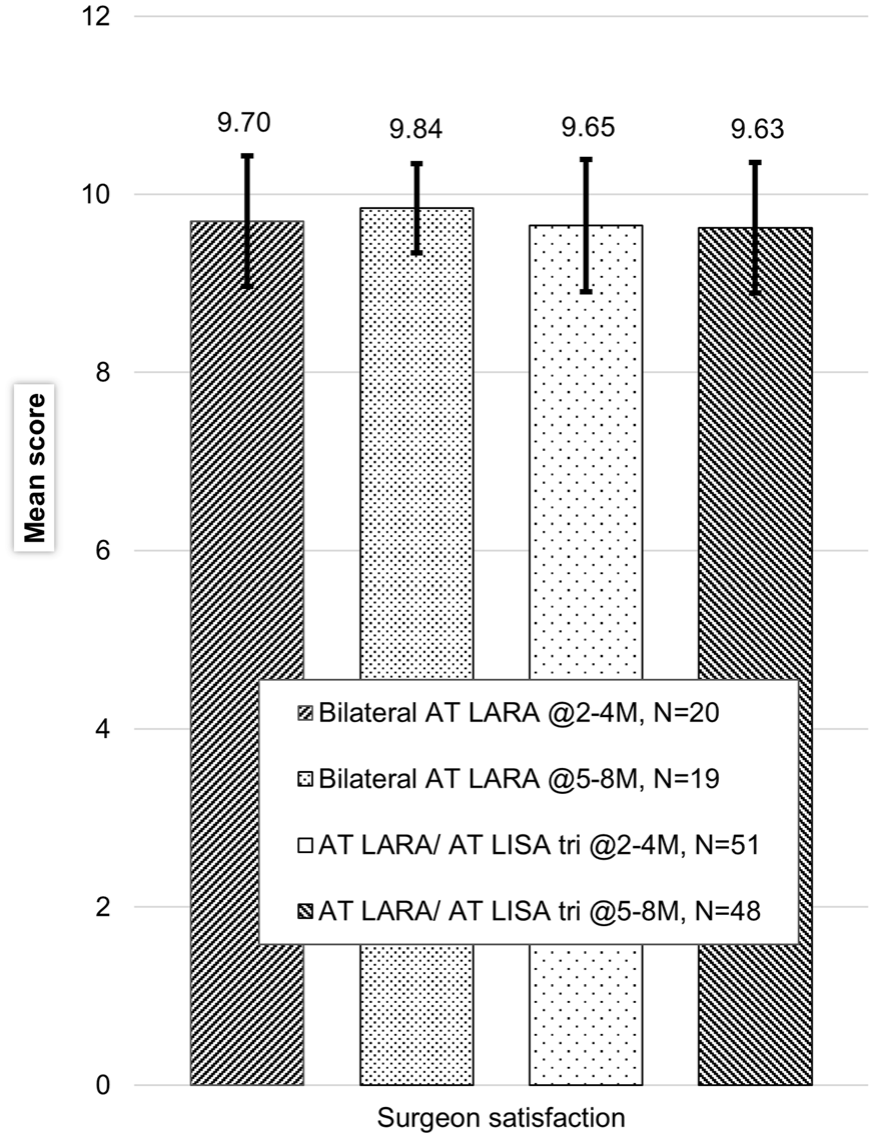
Postoperative surgeon satisfaction with visual outcomes

There were no differences in surgeon satisfaction rates between the bilateral or combined implantation groups in either implantation or ability to achieve target refraction. An in-house, non-validated surgeon satisfaction questionnaire was used. Figure 8 describes surgeon satisfaction levels when asked about patient vision.

## Discussion

Trifocal IOLs were introduced as a means to improve intermediate VA outcomes while keeping the advantages of other MFIOLs in near and distance vision; EDOF lenses attempt to provide a continuous range of uncorrected vision [1, 5, 9, 13, 14, 18]. The goal of using a combined implantation strategy with both a trifocal and an EDOF is to maximize the advantages of each lens’ optical designs [i.e., good near VA with the trifocal and superior intermediate VA (EDOF)] while producing fewer adverse photic phenomena [1, 9, 13, 14, 19].

In this study, we compared visual outcomes in patients who underwent cataract surgery or RLE and were implanted bilaterally with the AT LARA or with a combined implantation approach with the AT LARA/ AT LISA tri. Our results found good refractive stability and predictability through the last follow-up (5–8 months), with a mean spherical equivalent close to emmetropia in both groups. Overall, our study results now add to the literature and support a combined implantation approach for patients who want a continuous range of vision across all distances.

Some of our results differ from others in the literature, however, Song et al. [9] used the same combination of IOLs as our study did but reported a small percentage of combined implantation patients needed spectacles for near (4%) at the 6-month follow-up. Our study reported a higher percentage of patients who needed spectacles for near: 12/46 in the combined implantation AT LARA/AT LISA tri group (26.1%) vs. 12/18 in the bilateral AT LARA group (66.7%) needed spectacles at least “some of the time” at the 2–4 month follow-up, and 14/46 in the combined implantation AT LARA/ AT LISA tri group (30.4%) vs. 10/18 in the bilateral AT LARA group (55.6%) at the 5–8 month follow-up. Further, Song et al. reported none of their patients needed spectacles for intermediate or distance, whereas our results showed 4/46 in the combined implantation AT LARA/ AT LISA tri group (8.7%) and 1/18 in the bilateral AT LARA group (5.5%) needed spectacles “a little of the time” for distance; 5/46 in the combined implantation AT LARA/ AT LISA tri group (10.9%) and 4/18 in the bilateral AT LARA group (22.2%) needed spectacles at least “a little of the time” at the 2–4 month follow-up for intermediate vision. The differences between study results could be potentially explained by the different questionnaires used. While our study used the McAlinden quality of vision questionnaire and added questions about spectacle independence as well as surgeon preferences/satisfaction, Song et al. used the 25-item National Eye Institute’s Visual Function Questionnaire.

However, other studies that evaluated the AT LISA tri IOL had mixed outcomes compared to our results. Webers et al. [20] compared the uncorrected VA outcomes of 15 patients who were each bilaterally implanted with either the AT LISA tri IOL or the Tecnis Symfony EDOF IOL (Johnson & Johnson Vision, Jacksonville, FL, USA) in a randomized prospective study. Follow-up was at 1 week, 1 month, and 3 months. The authors concluded that there was no significant difference between groups in UNVA and UDVA; however, UIVA was significantly better in the bilateral EDOF group (mean UIVA was − 0.02 ± 0.03 logMAR in the EDOF group compared to 0.01 ± 0.03 logMAR in trifocal group, respectively [*P* = 0.047]). There were no differences in photic phenomena or patient satisfaction between groups.

Tarib et al. [14] used the same lenses as in our study to compare the visual outcomes at near, intermediate, and distance in 80 patients (*n* = 40 in each group) after 3 months of bilateral cataract surgery. The distance-corrected NVA was significantly better in the combined implantation group; however, there was no significant difference with regard to UDVA, UIVA, or uncorrected UNVA between the groups. In a separate study, Tarib et al. [13] compared visual outcomes at 3 months post-surgery in 42 patients (84 eyes) who were implanted with a diffractive MFIOL with low add power in the dominant eye and the AT LISA tri in the nondominant eye; they concluded the combined implantation approach provided good functional vision at all distances with the majority of patients not requiring spectacle use.

Acar et al. [1] used the same lenses as our study and reported similar UDVA and UNVA outcomes between the bilateral and combined implantation groups at 6 months postoperatively (*P* > 0.05). In their study, the combined implantation group showed significantly better intermediate visual outcomes at 60 cm and 80 cm than the bilateral group (*P* < 0.05). Further, the combined implantation group showed significantly better contrast sensitivity outcomes (photopic and mesopic) than the bilateral group (*p* < 0.05). Finally, there were no differences in defocus curves from 0.00 D to − 2.00 D, but significant differences from − 2.00 D to − 4.00 D.

Koo et al. [21] compared visual outcomes and patient satisfaction after bilateral implantation with an EDOF lens or two different combined implantation approaches with the Tecnis Symfony and bifocal IOLs. They also concluded that a combined implantation approach may provide better visual outcomes and higher patient satisfaction rates than bilateral implantation of the same lens.

Our results also found no significant difference between groups in either CDVA, UCDVA, UIVA, or corrected IVA at the last follow-up, although there was a statistically significant difference in UIVA at the 2–4 month follow-up (*P* = 0.048) favoring the bilateral approach. We also found both groups had comparable subjective rates and spectacle independence (at more than 90% for each variable), and acceptable objective vision outcomes.

This study provides longer-term follow-up (most others are at the 3-month postoperative time frame, with few at 6 months; this study evaluated patients at two time points: 2–4 months postoperative and 5–8 months postoperative). These findings suggest that refractive stability is achieved early, and initial visual results can be maintained over a longer period of time. This study’s results trend towards findings from McNeely et al. who evaluated visual outcomes from a combined implantation AT LARA/AT LISA tri over 12 months in patients (*N* = 58) who underwent either cataract or RLE surgery. In that study [11, 22], the combined implantation combination produced high functional vision and postoperative satisfaction, with statistically significant improvements in logMAR at 12 months compared to 1 month postop in uncorrected NVA (*P* = 0.008), uncorrected intermediate VA (*P* = 0.030), but not in UDVA (*P* = 0.323). This could be because patients achieved excellent distance vision within the first postoperative month and achieved early refractive stability. In the McNeely study, the mean binocular UDVA was–0.08 ± 0.07 logMAR at 1 month and – 0.09 ± 0.06 logMAR at 12 months [11, 22]. When McNeely et al. [22] analyzed refractive monocular outcomes, 58.6% of the eyes with the AT LARA were within 0.5 D at 1 and 12 months; 74.1% of the AT LISA tri eyes were within 0.5 D at 12 months, which declined slightly from the 79.3% of eyes that were within 0.5 D at 1 month. These percentages are slightly lower than others have reported: Schallhorn et al. [23] found a higher percentage of patients within 0.5 D (86.7%) after bilateral AT LARA implantation at 3 months.

It is important to note patient satisfaction in our study: 100% in the bilateral AT LARA group and 91.7% in the combined implantation AT LARA/AT LISA tri group would choose the same IOL again; these results are better than reported elsewhere. Rodov et al. [24] evaluated 200 eyes of 100 patients implanted with monofocal, monovision, EDOF (Tecnis Symfony), or trifocal (FineVision Micro F/POD F, PhysIOL, Liege, Belgium) to evaluate patient satisfaction, in addition to refractive outcomes, such as spectacle use and photic phenomenon. They reported better UNVA for patients implanted with the trifocal IOL than other IOLs. When asked about spectacle use, “never/rarely used” spectacles for intermediate vision was better for those implanted with the trifocal and EDOF lenses than the other two. Likewise, there was a low rate of postoperative halos/glare reported in the EDOF or trifocal lens groups (2% and 6%, respectively). When asked about IOL selection, 78% of those with the EDOF lens and 76% of those with the trifocal lens would choose the same IOL. The difference in lens choice may account for the substantial differences in patient satisfaction rates between these two studies.

Of interest, Kim et al. [16] noted patients with preoperative myopia needed spectacles for near even with combined implantation. While 40% of our patients underwent RLE (preoperative myopia was relatively low, averaging between − 0.25 D and − 0.46 D), 69% of our patients in combined implantation needed spectacles for near “none of the time” or “a little of the time.”

Our study also differs from most others that limited patient evaluations to those undergoing only cataract surgery. In our study, 60.6% of patients underwent cataract surgery, with the remaining patients undergoing RLE surgery. It is possible that the latter group was more motivated to achieve spectacle independence across all distances than the former group, which could have impacted the patient questionnaire outcomes.

This study is not without its limitations. Although unlikely because this study was prospective only after surgical implantation of the IOLs, it is possible that the lack of a standardized surgical procedure or phacoemulsification machine and preoperative prophylaxis regimens may have affected early postoperative refractive results. We believe this potential limitation is more than offset by the length of our follow-up and our reliance on real-world clinical settings. As noted earlier, 40% of our patients underwent RLE and not cataract surgery, which may also be a motivating factor to achieve spectacle independence. Yet our results aligned with McNeely et al., who also enrolled patients undergoing cataract or RLE surgery. Patient satisfaction is subjective and could be reflective of overall cataract surgery satisfaction. Patients choosing these lenses paid out-of-pocket, which could be a motivating factor and could have resulted in higher spectacle-independent expectations. The bilateral AT LARA group was counseled about the quality of distance, and intermediate vision that can be achieved with the lens but also that near vision outcomes would not be as robust, so expectations about near vision may have been mitigated. We also acknowledge the asymmetry in the number of patients included in each group (*n* = 20 in the bilateral group vs. *n* = 51 in the combination group). This 2:1 ratio may have accounted for the surprising finding that higher rates of halo were in the combination group, but we cannot state this emphatically. One hypothesis for the larger halo size could also be that AT LARA does not compensate for the average spherical aberration of the cornea which in theory could make the halo ring slightly thicker. Further, we did not have reading speed data from the bilateral group, so we were unable to make any generalizations or comparisons between groups, but believe future research should also include reading speed across the cohorts.

Finally, our study was halted early due to slow enrollment; and while unlikely, there is the possibility that a larger group of patients with bilateral implantation of the AT LARA lens may have altered results.

We recommend future research include a larger cohort, split evenly among bilateral AT LARA, combination AT LARA/AT LISA tri, and bilateral AT LISA tri.

## Conclusion

In this study, at the final 5–8 month postop time point, refractive outcomes were similar between a bilateral EDOF lens group and a combined implantation EDOF/trifocal lens group at distance, intermediate, and near. Patient satisfaction rates were extremely high for both groups. There were no new safety concerns raised in the combined implantation.

## Footnotes

1 Sample size calculations can be determined online from Sealed Envelope. Available from: https://www.sealedenvelope.com/power/continuous-noninferior/ [Accessed Mon Nov 05 2018].
